# Research on the influence mechanism of university-enterprise collaboration: Evidence From five southern coastal provinces in China

**DOI:** 10.3389/fpsyg.2022.929059

**Published:** 2022-08-16

**Authors:** Ximeng Chen, Yan Chen, Dongxue Li, Hao Dong

**Affiliations:** ^1^Institute of Vocational Education, Nanning Normal University, Nanning, China; ^2^China-ASEAN Vocational Education Research Center, Nanning, China; ^3^Guangxi Vocational Education Development Research Center, Nanning, China; ^4^Nanning University, Nanning, China; ^5^Institute of Land Engineering and Technology, Shaanxi Provincial Land Engineering Construction Group Co., Ltd., Xi’an, China

**Keywords:** collaborative innovative interface management, interface connection mechanism, collaborative innovation performance, partial least squares structural equation modeling, fuzzy-set qualitative comparative analysis

## Abstract

Taking university-enterprise collaborative innovation in five southern coastal provinces of China as subjects, empirical research is implemented by constructing a theoretical model of the effects of interface resource integration, interface conflict management, interface connection mechanisms, and enterprise absorptive capacity on the university-enterprise collaborative innovation performance with the partial least squares structural equation modeling and fuzzy-set qualitative comparative analysis. A total of 245 valid questionnaires were collected from five coastal provinces in south China. The research results show that the interface resource integration, interface connection mechanisms, and enterprise absorptive capacity has direct significant positive impacts on the collaborative innovation performance. Interface conflict management has no significant impact on school-enterprise collaborative innovation performance. Moreover, the interface connection mechanism acts as an intermediary. Therefore, it is suggested that university-enterprise should integrate resources with complementing, interacting, and sharing resources; construct the profit and risk-sharing mechanism, communication and trust mechanism, and organizational learning mechanism.

## Introduction

University-enterprise collaborative innovation is a systematic project of joint action by both sides, which aims to integrate the resources of enterprises and universities and improve the innovation ability of enterprises (Wang et al., 2021). The key to collaborative innovation between universities and enterprises is for both sides to break through their respective boundaries and form a cross-institutional interaction area to promote the interaction of innovation subjects and enhance innovation performance (Zhuang et al., 2021). However, the interface management problems caused by the scattered innovation resources and barriers among the participating subjects make it difficult to integrate the collaborative innovation resources, coordinate the interests and sustain the cooperation. How to carry out the interface management of university-enterprise collaborative innovation is a hot issue in the field of collaborative innovation research at present (Villani et al., 2017).

Interface as an interdisciplinary term first appeared in engineering and technology to describe the interfaces between various mechanical components (assemblies.) In the 1970s, it was introduced into the management discipline to describe the connections between functions or between organizations in management activities. For example, it concentrated on the study of enterprise research and development (R&D) and supplier interfaces, production interfaces, and marketing interfaces. Souder and Chakrabarti found that when there are serious problems in the management of the R&D a marketing interface, it leads to the failure of 68% of R&D projects in terms of commercialization (Souder and Chakrabarti, 1978). The existing literature on interface management research now covers all aspects of strategic enterprise management (Zhao et al., 2017). With the advent of the digital age, innovation models are showing increasingly open characteristics and the literature on interface management research is growing (Waxman et al., 1999), and interface management has become an important strategy to reduce the risk of collaborative innovation and R&D costs. It is an important way for enterprises to acquire external knowledge, resources, and capabilities. In recent years, studies on the interface management of university-enterprise collaborative innovation have started to emerge (Etzkowitz and Leydesdorff, 2000). Relevant studies have found that “zero” interface, “stacked” interface and “full” interface, or process interface, task interface and organizational interface have been formed due to the disengagement, participation and interaction of knowledge diffusion and flow between universities and enterprises. The collaborative innovation performance is affected by the lack of interface management functions and the ineffective resolution of cooperation conflicts.

The university-enterprise collaborative innovation interface mainly consists of cross-border players (universities, enterprises, and technology intermediaries), media (technology, capital, information, and other media), and linkage mechanisms (mechanisms of benefit distribution, information exchange, knowledge learning, etc.; Liu et al., 2019). The interface management of university-enterprise collaborative innovation is to achieve the goal of university-enterprise collaborative innovation by designing a reasonable connection mechanism, actively intervening in the interaction relationship, resolving interface conflicts, overcoming the practical difficulties of scattered innovation resources and closed innovation activities, and promoting cross-interface interaction and interaction between universities and enterprises (Xu et al., 2018). The literature has provided a theoretical basis for the research on the management of university-enterprise collaborative innovation interfaces (Rajalo and Vadi, 2017). However, there is still room for improvement in the research on the interface management of university-enterprise collaborative innovation. First, the existing studies focus on the interface management between different functional departments within enterprises, but less on the interface management of university-enterprise cooperation. Some studies on the interface management of university-industry cooperation have been conducted mainly from the perspective of mutual independence of enterprises, universities, and research institutes, lacking an integrated theoretical model and data support. Barnes et al. (2002) examines what kind of R&D focus inside the firm will improve or reduce the benefits of R&D collaborations with universities; Bruneel et al. (2010) explored ways to reduce barriers to cooperation between universities and enterprises by the perspective of universities; Arvanitis et al. (2008) empirically examines the factors that determine the propensity of Swiss scientific institutions to participate in broad knowledge and technology transfer activities of private firms at the level of individual institutes or sectors. Secondly, the existing studies have ignored the role of soft elements such as interface operation mechanism and interface conflict management in the process of university-enterprise collaborative innovation. Thirdly, the factors influencing the interaction of innovation agents in the collaborative innovation interface and their paths of action are still in a “black box” state, and the questions that need to be further explored include: (1) Do the interface organizational resources, conflict management, and the absorptive capacity of enterprises have a positive impact on the performance of university-enterprise collaborative innovation? (2) Do Interface connection mechanisms play a mediating role in the integration of interface resources, conflict management, enterprise absorptive capacity, and collaborative innovation performance? (3) What is the realization path of university-enterprise cooperation? Since the existing literature lacks in-depth and systematic interpretation and data support. This paper intends to explore the interaction between organizational resources, conflict management, linkage mechanism, absorptive capacity of enterprises and university-enterprise collaborative innovation performance in the university-enterprise collaborative innovation interface by constructing a structural equation model, and explore the path to improve university-enterprise collaborative innovation performance based on the survey data of technology-based SMEs in five southern coastal provinces in China.

## Theoretical analysis and research hypotheses

### Degree of integration of resources between interface organizations

Sirmon et al. (2007) argue that resource integration is the process by which an organization acquires needed resources and reconfigures the original resource system to form a new core resource system and capabilities. The effectiveness of resource management in interface organizations depends mainly on the degree of inter-organizational resource complementarity, the degree of sharing and the degree of team (human resource) interaction (Boudreau et al., 2003).

First, the degree of complementarity of inter-organizational resources is an important prerequisite for integrating innovation resources. The basic research capability owned by universities and the technology development capability owned by enterprises, etc. are important complementary resources between organizations (Micheli et al., 2014). Secondly, the key to achieving the effective integration of resources between organizations from fragmentation is to realize the sharing of resources between organizations. For example, the degree of sharing of pilot equipment owned by enterprises and R&D experimental equipment owned by universities in the process of university-enterprise cooperation, as well as the degree of sharing of R&D information and market information, all reflect the degree of convergence and utilization of organizational resources (Moshtari, 2016). Third, the team is an important human resource in the organization. Only team interaction can facilitate the flow of knowledge between different institutions and enhance the performance of science and technology innovation. Relevant studies show that collaboration, cohesion, task interaction and innovation performance of R&D teams show significant positive correlation (Biermann, 2008). Based on this, the following hypothesis is proposed.

**Hypothesis (H1).** The degree of organizational resource integration is positively related to university-enterprise collaborative innovation performance.

### Interface conflict management

Jehn (1997) argues that conflict has become a significant constraint on organizational performance. Because universities and enterprises are clearly heterogeneous institutions, there are more obvious conflicts of goals, interests and cultures. Universities favor the academic nature of research results, while enterprises pursue the commercial nature of research results; enterprises undertake collaborative innovation to obtain intellectual property rights to gain technological advantages, while universities reduce R&D risks by obtaining funding (Raudla et al., 2015). Due to the different positions held by the two parties, they will have some obvious conflicts in the general objectives of collaborative innovation, such as conflict of interest (Overton and Lowry, 2013). It manifests itself in the inconsistent understanding of the valuation of the university’s intellectual property; the disagreement on the treatment of the ownership of intellectual assets generated in the process of collaboration. In terms of benefit distribution, universities hope that knowledge innovation can be valued as shares, while enterprises may regard university researchers as “temporary workers” rather than “shareholders”; enterprises hope to pay university researchers’ R&D costs in installments in order to spread financial risks. However, due to the uncertainty and risk factors of technological innovation, university researchers want to be paid more by the enterprise when the project starts. In addition, due to the difference in organizational culture, universities and companies tend to use “self-referential standards” to think about their own values, management philosophy and organizational practices, which may lead to insufficient effective communication and lack of trust, resulting in inefficient cooperation. Tjosvold et al. (2003) believes that the adoption of appropriate conflict management approaches can effectively mitigate the negative impact of destructive or dysfunctional conflicts on organizations. The important function of collaborative innovation interface management in a university-enterprise is to remove barriers in the interface and resolve conflicts between different innovation agents. Based on this, the following hypothesis is proposed.

**Hypothesis (H2).** Interface conflict management is positively related to university-enterprise collaborative innovation performance.

### Enterprise absorption capacity

Cohen and Levinthal (1990) argue that the key to enterprise development lies in the ability to identify, acquire, digest, and utilize new external knowledge from practice, i.e., absorptive capacity. Existing studies on enterprise innovation performance generally take the absorptive capacity of enterprises as an important independent variable to study its relationship with enterprise innovation performance, and find that the absorptive capacity of enterprises has a significant positive impact on the integration of enterprise knowledge and innovation performance (Najafi-Tavani et al., 2016), but few studies have investigated the relationship between the absorptive capacity of enterprises and the collaborative innovation performance of university-enterprise. Absorbing external knowledge resources is an important way for technology-based enterprises to improve their R&D capabilities and innovation performance. In a collaborative innovation environment, the stronger the absorptive capacity of an enterprise, the better its ability to acquire and utilize knowledge from the university, the more effective it is in developing new products and predicting the commercialization potential of R&D results. Therefore, there is a logical causal relationship between absorptive capacity and university-enterprise collaborative innovation performance. Based on this, the following hypothesis is proposed.

**Hypothesis (H3).** Enterprise absorptive capacity is positively related to university-enterprise collaborative innovation performance.

### Interface connection mechanisms

Zhang et al. (2022) found that the linkage mechanism between enterprises is different from the administrative mechanism of internal organizational sections and the price mechanism of market transactions; it is a linkage mechanism of benefit sharing and risk sharing based on information and resource sharing. University-enterprise cooperation is an evolutionary process in which both organizations interact with each other in the interface of personnel, knowledge, technology, information and capital, reflecting a cross-organizational linkage. To strengthen organizational innovation capability (Camisón and Villar-López, 2014), we need to rely on the linkage mechanism of collaborative innovation interface to coordinate, integrate and activate organizational resources, resolve conflicts and contradictions, and thus enhance innovation performance (Tong and Han, 2021). Some researchers have conducted empirical evidence with high-tech development zones and university science and technology parks in Jiangsu province, and found that the connection mechanism constructed by innovation subjects helps to improve innovation performance. Some studies also confirmed that collaborative innovation mechanism has a significant positive impact on enterprise innovation activities (Huang and Li, 2017). Based on this, the following hypothesis is proposed.

**Hypothesis (H4).** The Interface connection mechanisms is positively related to university-enterprise collaborative innovation performance.

Chen and Tao (2021) research also found that the innovation environment does not have a significant impact on enterprise collaborative innovation participation directly, but relies precisely on the mediating and bridging role of collaborative innovation mechanism to influence enterprise collaborative innovation participation. To achieve good cooperation effect among cross-organizations, it is necessary to conduct cross-sector and inter-institutional information communication and exchange, which requires good resource sharing and information communication between universities and enterprises. Based on this, the following hypothesis is proposed.

**Hypothesis (H5).** Interface connection mechanisms play a mediating role in inter-organizational resource integration and university-enterprise collaborative innovation performance.

To address the problems of conflicting goals, interests and cultures in the process of collaborative innovation, we need to play the role of “benefit distribution, communication and coordination” mechanisms to resolve conflicts and promote cooperation (Mishra and Mishra, 2009). Based on this, the following hypothesis is proposed.

**Hypothesis (H6).** Interface connection mechanisms play a mediating role in interface conflict management and university-enterprise collaborative innovation performance.

Enterprises have a certain absorptive capacity, but they need to rely on organizational learning mechanisms in order to identify, acquire and digest, and apply the university’s innovative knowledge and carry out technological innovation activities (Kim and Rhee, 2018). Based on this, the following hypothesis is proposed.

**Hypothesis (H7).** Interface connection mechanisms play a mediating role in enterprise absorptive capacity and university-enterprise collaborative innovation performance.

### Conceptual model

**FIGURE 1:**
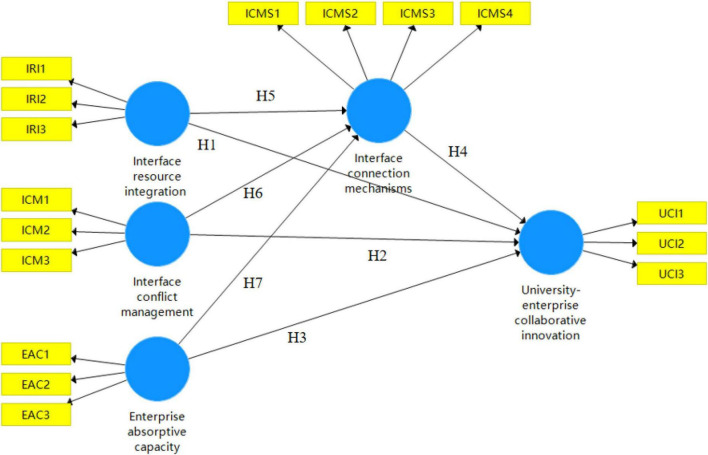
Research framework.

Based on the synthesis of many research results, combined with the preliminary survey research and group interviews, the framework of this study was determined (see Figure 1), in which the independent variables are organizational resource integration, organizational conflict management, enterprise absorptive capacity and Interface connection mechanisms of the collaborative innovation interface, and the dependent variable is collaborative innovation performance. This study attempts to explore the mediating role of the Interface connection mechanisms.

## Materials and methods

### Sampling and data collection

In this study, technology-based SMEs in the five southern coastal provinces that carry out collaborative innovation with universities were selected as the subjects of empirical research. Mainly by contacting universities and local government management departments of science and technology innovation and technology transfer to understand the situation of local enterprises, then questionnaires were distributed in the field and by email, and the questionnaires were sent to the heads of technology departments or top managers of enterprises. A total of 325 questionnaires were distributed and 269 were returned, with a return rate of 82.8%. The questionnaires were then screened according to whether the questionnaires were filled out in a standardized manner and whether the enterprises carried out collaborative innovation as important criteria. 245 valid questionnaires were screened out, with a valid rate of 75.4%. The characteristics of the enterprises are shown in Table 1. The analyzed enterprises all carried out collaborative innovation with universities, which is representative in terms of the structural characteristics of the sample and meets the requirements of this study.

**TABLE 1:** Distribution of questionnaires on entrepreneurship of rural farmers in the five western provinces.

Characteristics	Samples	Percent (%)
**Industry**		
Service activities and utilities	62	25.3
Manufacturing	59	24.1
ICT	55	22.4
Trade and retail	31	12.7
Agri-food	19	7.8
Financial, insurance and banking activities	14	5.7
Others (e.g., R&D, Construction, Transportation, Real Estate)	5	2.0
**Employee**		
1–99	39	15.9
100–299	78	31.8
300–499	41	16.7
500–999	87	35.5
**Enterprise age**		
<5 years	45	18.4
5–9 years	114	46.5
10–24 years	62	25.3
>25 years	24	9.8

### Measures

Combined with the theoretical framework model in Figure 1, the measurement of the management factors of the university-enterprise collaborative innovation interface in this study mainly includes the degree of inter-organizational resource integration, the degree of organizational conflict resolution, the organizational linkage mechanism, the absorptive capacity of enterprises, and the collaborative innovation performance. To ensure the reliability and validity of the measurement instruments, the variables involved in this study were measured mainly by scales that have been used in published authoritative literature, and a pre-survey of questionnaires was conducted in some technology-based SMEs before the questionnaires were formalized, and the opinions of university collaborative innovation experts were also sought to assess the rationality of the questionnaire design. The Likert 7-level scale is used to measure the above variables, and the measurement range is from “very dissatisfied” to “very satisfied” corresponding to the numbers “1” to “7.” Table 2 lists the variables and their measurement methods used in this study.

**TABLE 2:** Survey variables and measures.

Variable	Measurement item	Sources
Interface resource integration	Degree of resource complementarity	Sirmon et al., 2007
	Degree of resource sharing	
	Degree of team interaction	
Interface conflict management	Conflict of goals	Jehn, 1997
	Conflict of interest	
	Conflict of culture	
Interface connection mechanisms	Benefit distribution mechanism	Lee et al., 2010
	Communication and trust mechanism	
	Organizational learning mechanism	
Enterprise absorptive capacity	Knowledge recognition	Cohen and Levinthal, 1990
	Knowledge acquisition	
	Knowledge digestion	
	Knowledge application	
University-enterprise collaborative innovation	New product development cycle	Rajalo and Vadi, 2017
	New product development costs	
	New product development success rate	

## Analysis result

### Evaluation of measurement model

The results showed that the measurement model satisfies all general requirements (see Table 3). First, all the standardized factor loadings of all the first-order and second-order constructs are above the minimum value of 0.756 (Fornell and Larcker, 1981). Second, the Cronbach’s alpha scores ranged between 0.749 and 0.924 while the composite reliability scores ranged between 0.857 and 0.950 which are above the recommended value of 0.70 indicating adequate construct validity. In addition, all the constructs have an AVE value above 0.50, suggesting that latent variables achieved convergent validity. Finally, this study follows three approaches to assess the discriminant validity, i.e., (1) Fornell-Larcker criterion, (2) cross loading, and (3) the heterotrait-monotrait ratio of correlations (HTMT).

**TABLE 3:** Reliability and validity.

Variable	Item	Convergent validity			Cronbach’s alpha	Multicollinearity
		
		Cross loadings	Composite reliability	AVE		VIF
Interface resource integration	IRI1	0.846	0.875	0.701	0.786	1.689
	IRI2	0.843				1.666
	IRI3	0.822				1.588
Interface conflict management	ICM1	0.824	0.857	0.666	0.749	1.471
	ICM2	0.824				1.549
	ICM3	0.800				1.485
Enterprise absorptive capacity	EAC1	0.756	0.874	0.699	0.784	1.402
	EAC2	0.873				1.974
	EAC3	0.873				1.877
Interface connection mechanisms	ICMS1	0.846	0.906	0.706	0.861	2.113
	ICMS2	0.857				2.208
	ICMS3	0.821				1.945
	ICMS4	0.836				1.841
University-enterprise collaborative innovation	UCI1	0.911	0.950	0.863	0.921	2.990
	UCI2	0.946				4.173
	UCI3	0.931				3.477

The correlation matrix in Table 4 shows that for each pair of constructs, the AVE square root of each construct is higher than the absolute value of their correlation (Fornell and Larcker, 1981). The results of cross loading show that all items are loaded higher on their respective constructs than on the other constructs and the cross-loading differences are much higher than the suggested threshold of 0.1. In all cases the HTMT values are below the threshold of 0.85. These results conenterpriseed that the discriminant validity is present in this study.

**TABLE 4:** Discriminant validity – Fornell-Larcker Criterion and Heterotrait – Monotrait ratio.

Variables	Mean	S.D	1	2	3	4	5
1. .Interface connection mechanisms	4.76	1.12	**0.840**	0.586	0.744	0.784	0.749
2. .Interface conflict management	5.05	1.22	0.477**	**0.816**	0.587	0.651	0.525
3. .Enterprise absorptive capacity	4.83	1.20	0.623**	0.446**	**0.836**	0.769	0.666
4. .Interface resource integration	4.94	1.12	0.647**	0.501**	0.605**	**0.837**	0.732
5. .University-enterprise collaborative innovation	5.1	1.05	0.673**	0.438**	0.566**	0.625**	**0.929**

Bold diagonal entries are the square root of AVEs. **p < 0.01.

### Evaluation of symmetrical modeling

This study followed Hair et al. (2012) to estimate the structural model. First, the results show minimal collinearity in the structural model as all VIF values are far below the common cutoff threshold of 5 to 10 Hair et al. (2012). Second, following the rules of thumb, the *R*^2^ values of ICMS (0.517) and UCI (0.529) exceed the minimum value of 0.10 recommended by Hair which is a satisfactory level of predictability as shown in Table 5. Similarly, results from blindfolding with an omission distance of six yield *Q*^2^ values well above zero (Table 5). This supporting the model’s predictive relevance in terms of out-of-sample prediction. Further analysis of the composite-based standardized root mean square residual (SRMR) yields a value of 0.062, which conenterprises the overall fit of PLS path model (Henseler et al., 2014). Applying the bootstrapping procedure (5,000 bootstrap samples; no sign changes) provides the *p*-values as well as the corresponding 95% bias-corrected and accelerated bootstrap confidence intervals (Table 5). The empirical results support the vast majority of hypothesized path model relationships among the constructs. The significance testing results of the structural model path coefficients confirm that Enterprise absorptive capacity → Interface connection mechanisms (H7:β = 0.333, *p* < 0.000), Enterprise absorptive capacity → University-enterprise collaborative innovation (H4:β = 0.140, *p* < 0.035), Interface conflict management → Interface connection mechanisms (H6:β = 0.141, *p* < 0.023), Interface conflict management → University-enterprise collaborative innovation (H2:β = 0.06, *p* < 0.249), Interface connection mechanisms → University-enterprise collaborative innovation (H3:β = 0.390, *p* < 0.000), Interface resource integration → Interface connection mechanisms (H5:β = 0.374, *p* < 0.000), Interface resource integration → University-enterprise collaborative innovation (H1:β = 0.258, *p* < 0.004; Table 5).

**TABLE 5:** Significant testing results of the structural model path coefficients.

	Path coefficient	*t*-value	*P*-value	95% BCa confidence interval	Conclusion
Enterprise absorptive capacity → Interface connection mechanisms	0.333	5.849	0.000	(0.224,0.448)	H7 supported
Enterprise absorptive capacity → University-enterprise collaborative innovation	0.140	2.105	0.035	(0.013,0.276)	H4 supported
Interface conflict management → Interface connection mechanisms	0.141	2.274	0.023	(0.021,0.265)	H6 supported
Interface conflict management → University-enterprise collaborative innovation	0.06	1.153	0.249	(-0.039,0.163)	H2 not supported
Interface connection mechanisms → University-enterprise collaborative innovation	0.390	5.689	0.000	(0.252,0.525)	H3 supported
Interface resource integration → Interface connection mechanisms	0.374	5.323	0.000	(0.238.0.509)	H5 supported
Interface resource integration → University-enterprise collaborative innovation	0.258	2.854	0.004	(0.065,0.408)	H1 supported

SRMR composite model = 0.062. R^2^_ICMS_ = 0.517; Q^2^_ICMS_ = 0.354. R^2^_UCI_ = 0.529; Q^2^_UCI_ = 0.443.

### Fuzzy set-quality comparative analysis approach

Fuzzy-set qualitative comparative analysis uses Boolean algebra to generate combinations of causal conditions leading to an outcome. Central to the fsQCA approach are the calibration procedure and the truth table analysis. The calibration is a transformation process consisting in converting conventional measures into fuzzy sets. The truth-table analysis produces three different solution terms: (1) complex, (2) parsimonious, and (3) intermediate (Rihoux and Ragin, 2009). Fiss (2011) propose the mix of the last two solutions to bring out core and peripheral conditions, associated with the outcome of interest. Core conditions are solutions belonging to both parsimonious and intermediate that show a strong causal relationship with the outcome, whereas peripheral conditions are solutions appearing only in the intermediate solutions and presenting a weaker relationship with the outcome.

#### Calibration procedure

Fuzzy set calibration is a key operation in the fsQCA. Calibration refers to the process of assigning set membership to cases and establishing a relationship between variable values and fuzzy set membership by locating points. Combined with the actual situation of the research data, this study selected the 95% quantile, median (50%), and 5% quantile of the sample data as the three calibration points of full membership, crossover point, and full non-membership of the result and conditional variables. The results and calibration information for each conditional variable are listed in Table 6.

**TABLE 6:** Calibration positioning points of case variables.

Variables		Locating point		
		
		Full membership	Crossover point	Full non-membership
Outcome variables	UCI	7	5	3.667
Conditional variables	IRI	6.333	5	3.667
	ICM	6.667	5	3.333
	EAC	6.6	4.667	3.333
	ICMS	6.25	4.75	3.25

#### Fuzzy-set qualitative comparative analysis solution

Before the condition configuration analysis, it is necessary to check the “necessity” of each condition individually. The necessary condition leads to the occurrence of the result, but its existence does not guarantee the inevitable existence of the result. Consistency is an important test standard for the necessary conditions. When the consistency score is greater than 0.90, this condition is necessary for the result (Ragin, 2006). The above calibrated fuzzy value is input into the fsQCA software for necessary condition analysis, and the results are summarized in Table 7.

**TABLE 7:** Calibration positioning points of case variables.

	High performance	
	
Conditional variable	Consistency	Coverage
ICM	0.810645	0.769502
∼ICM	0.557338	0.604266
IRI	0.783548	0.828162
∼IRI	0.589677	0.572681
EAC	0.791129	0.779933
∼EAC	0.555483	0.577755
ICMS	0.817016	0.826481
∼ICMS	0.558064	0.565267

#### Fuzzy-set qualitative comparative analysis solution

The sufficiency analysis of conditional configuration explores whether the set represented by the configuration composed of multiple conditions is a subset of the result set from the perspective of set theory. When using fsQCA 3.0 software for configuration analysis, the relevant parameters should be set according to the research needs. In this study, the original consistency threshold was set to0.80, PRI consistency threshold was set to 0.5, and the case frequency threshold is set to 1. In software fsQCA Ver.3.0, three solutions were generated using the standard analysis program: complex solution, intermediate solution, and reduced solution. The core conditions of each solution can be identified by comparing the nested relationship between the intermediate and reduced solutions.

We use the QCA results proposed by Ragin to analyze the configuration of enterprise performance. The results are listed in Table 8, in which each column represents a possible conditional configuration. The results show that these three paths lead to a high-level of enterprise performance. Solution 1: interface connection mechanisms, interface resource integration, and interface connection mechanisms, the consistency is 0.89004, and the coverage is 0.597324. This result proves the impact of interface connection mechanisms, interface resource integration, and interface connection mechanisms on university-enterprise collaborative innovation performance. Solution 2: ∼interface connection mechanisms, interface resource integration, enterprise absorptive capacity, and interface connection mechanisms. The consistency was 0.920733 and the coverage was 0.618294. This result further proves the impact of ∼interface connection mechanisms, interface resource integration, enterprise absorptive capacity, and interface connection mechanisms on university-enterprise collaborative innovation performance. Solution 3: interface resource integration, enterprise absorptive capacity, and interface conflict management. The consistency was 0.906018 and coverage was 0.618504.

**TABLE 8:** Sufficiency analysis of conditional configuration (performance).

	Path		
	
Conditional configuration	Configuration 1	Configuration 2	Configuration 3
ICM	●	⊗	●
IRI	●	●	●
EAC		●	●
ICMS	●	●	
Raw coverage	0.597324	0.618294	0.618504
Unique coverage	0.0391102	0.0600797	0.0602894
Consistency	0.890040	0.920733	0.906018
Solution coverage		0.785985	
Solution consistency		0.861784	

#### Robustness test

We used standard methods to conduct a robust analysis of QCA results. The commonly used methods are: Adjust the calibration threshold, change the consistency threshold, add or delete the shell, change the frequency threshold, and add other conditions. Method 1: Referring to the practice of Fiss, the robustness test is carried out by adjusting the crossing point of calibration. Specifically, the crossing point is adjusted from 0.5 to 0.55. The number of configurations and the neutral permutations with the same core conditions but different edge conditions all changed slightly, but the changes were not enough to support meaningful and completely different substantive interpretation method 2. Referring to the set relation and quasi-sum difference of configurations proposed by Schneider and Wagemann (Schneider and Wagemann, 2012) as the judging criteria, this paper reduced the consistency threshold from 0.8 to 0.75 and found that the research configurations were still supported. Therefore, the research conclusions of this paper are still robust.

## Conclusion and discussion

### Conclusion

In this study, the following findings were obtained by exploring the effects of inter-organizational resource integration degree of collaborative innovation interface, interface conflict management, and enterprise absorptive capacity on university-enterprise collaborative innovation performance, and the mediating role of interface connection mechanism between them.

In the collaborative innovation interface between universities and enterprises, the path of “interface conflict management → interface connection mechanism → collaborative innovation performance” is formed, and the interface connection mechanism plays a fully of intermediary role. Therefore, it is important to build a reasonable linkage mechanism, especially the benefit distribution mechanism, communication and trust mechanism, to resolve the interface conflict.

In the collaborative innovation interface between universities and enterprises, the path of “enterprises’ absorptive capacity → interface linkage mechanism → collaborative innovation performance” is formed, and the interface linkage mechanism plays a part mediating role. This indicates that the construction of reasonable connection mechanism, such as organizational learning mechanism, is of great value to bring into play the absorptive capacity of enterprises, timely absorb the innovation knowledge of universities, and promote the transfer of scientific and technological achievements.

The degree of integration of organizational resources at the interface and the absorptive capacity of enterprises all have significant and direct positive effects on enhancing the performance of university-enterprise collaborative innovation, while interface conflict management does not have a significant positive effect on innovation performance. Therefore, targeted enhancement of the degree of complementarity of organizational resources, the degree of mutual sharing of resources and the degree of interaction of human resources to integrate resources; resolving goal conflicts, interest conflicts and cultural conflicts of university-enterprise collaborative innovation through the interface connection mechanism to promote the deep integration between universities and enterprises; targeted efforts on the links of identification, acquisition, digestion and application of university knowledge by enterprises will help to improve university-enterprise collaborative Innovation performance.

### Theoretical contribution

Based on the above findings, this study proposes that the integration of inter-organizational resources should be emphasized, interface conflict management should be carried out by fully leveraging the mediating role of interface connection mechanism, and the university-enterprise collaborative Innovation performance should be enhanced by strengthening the absorptive capacity of enterprises.

First. Establish an innovative resource integration model based on complementarity, interaction and mutual sharing. Focusing on the common goal of collaborative innovation, with the complementarity of resources as the premise, the interaction of human capital as the key, and the sharing of various types of innovation resources as the focus, we fully gather the advantageous resources of each innovation body to achieve complementary mutual use. Integrate resources with the premise of complementarity of resources (Chen et al., 2017). The complementarity of innovation resources of universities and enterprises is an important internal driving force for both sides to carry out cooperation. Before carrying out substantive cooperation, the university and enterprises conduct mutual research and communication, systematically sort out the existing resources and inform each other, so that both sides know in advance the resources that can be shared and thus improve the sharing rate and efficiency of using resources in a targeted manner. Promote the integration of resources by taking the interaction of human resources as fundamental (Monforti et al., 2014). Human resources are the executors of innovation activities and the carriers of other resources, especially knowledge resources. Through the interaction and exchange of cooperative teams, it is conducive to the sharing of resources owned by each innovation body, form a situation of small knowledge potential difference and high innovation performance of university-enterprise cooperation (Shang et al., 2021). Build an “innovation resource pool” to fully integrate all kinds of innovation resources. The process of collaborative innovation requires material and economic resources, as well as various information and policy support. Universities and enterprises can support collaborative innovation by building “resource pools,” and universities and enterprises can give full play to the role of industry associations, public innovation platforms, and relevant technology intermediaries to establish channels and bridges for absorbing various innovation resources. For example, universities can attract high-level talents to join the university-enterprise cooperation team by virtue of the influence of advantageous characteristic disciplines, and attract venture capital to fund the incubation of projects.

Second. The mechanism of benefit sharing and risk sharing is to coordinate and resolve the conflict of interests and risk crisis between universities and enterprises across organizations, and seek a balance of mutual interests. Adhering to the basic principle of “benefit sharing and risk sharing,” the university-enterprise collaborative innovation benefit sharing and risk sharing mechanism is established on the premise of clear ownership of intellectual property rights, based on a reasonable benefit distribution method and guaranteed by a perfect benefit distribution system, which ensures mutual benefits and sustainable stability of cooperation from multiple aspects, and Manages intellectual property issues and how to deal with knowledge (Mahfoudh et al., 2021). For the characteristics of university-enterprise collaborative innovation, establish a communication and trust mechanism based on system guarantee and process interaction. Establish a trust mechanism based on system. At the early stage of university-enterprise collaborative innovation, it is difficult to establish trust relationship, so we need to rely on the system to guarantee the establishment of trust relationship. By formulating systems such as integrity system, information exchange system and technical confidentiality, the cooperative behavior of members is regulated and violations are punished to prevent opportunistic behavior and deceitful behavior of members. Establish trust based on process interaction. Continuous information communication is an important source for partners to generate trust. It can address the increasing formalization and monitoring of the university’s operational relationships that can lead to conflict and mistrust between parties attempting to maintain the autonomy of their organizations in the face of increasing interdependence (Mahfoudh et al., 2021). To strengthen the construction of information exchange channels, reasonable matching can enhance the interdependence of knowledge and organization formed between partners, which in turn affects the synergy of cooperation (Cai and Zhang, 2012), on the one hand, develop a regular exchange system between senior management and managers. The top level and managers are often at the core of the interface, and their demonstration role plays an important demonstration role in promoting inter-organizational communication and cooperation, resolving conflicts and enhancing trust, so formal communication channels can be established.

### Management implications

Although universities and enterprises are both knowledge sources, the strong R&D capability of the cooperating universities and the insufficient absorption capability of the cooperating enterprises will make it difficult to absorb, digest, apply and transfer the innovative knowledge of universities into commodities in a timely manner. The establishment of efficient organizational learning mechanism can help the diffusion of knowledge in the process of university-enterprise cooperation and promote the integration and absorption of knowledge by enterprises. Exploratory learning and excavation learning mechanisms of organizations proposed by March have insights for the establishment of organizational learning mechanism of university-enterprise cooperation. On the one hand, exploratory learning mechanism is established. Exploratory learning mainly emphasizes the organization’s search for external knowledge to acquire new knowledge and discover new business opportunities in order to form the organization’s competitiveness. Due to the influence of organizational inertia, existing technologies and behavioral patterns, enterprises’ willingness to acquire knowledge through exploratory learning will not be strong. Therefore, enterprises are encouraged to cooperate with universities to conduct innovation experiments, and in the process of research experiments, both parties establish exploratory learning mechanisms by jointly tackling R&D challenges. On the other hand, an exploratory learning mechanism is established. Excavative learning means that universities and enterprises have defined R&D tasks and use the organization’s existing knowledge and skills to improve products and services in order to enhance innovation performance. For example, enterprises provide a certain technology to directly help university researchers to complete the subsequent development of the product; or university researchers provide a certain innovative knowledge to directly help enterprises to improve the quality of a certain product. Both learning mechanisms are needed for university-enterprise collaborative innovation and should be used at the right time according to the characteristics of the R&D tasks jointly undertaken by universities and enterprises. For example, exploratory learning should be favored when the university-enterprise collaborative organization needs to acquire new knowledge from outside the organization, and exploratory learning should be favored when it needs to use existing knowledge to improve a product.

### Limitations and future research

This study solved some gaps in the literature, but there are still some limitations that need further discussion. First, the data used in this study was collected only in China. Since different research Settings and other samples may lead to different findings, researchers may wish to use data from other emerging economies to examine various entrepreneurial actions. Secondly, this study can use the fuzzy set qualitative comparative analysis method to study the antecedents of university-enterprise cooperation path, and obtain the path of university-enterprise cooperation innovation performence.

## Data availability statement

The original contributions presented in this study are included in the article/supplementary material, further inquiries can be directed to the corresponding author.

## Author contributions

HD, DL, and XC: methodology and software, formal analysis, resources, data curation, and writing—review and editing. HD and DL: investigation. YC: writing—original draft preparation, supervision, and project administration. All authors read and agreed to the published version of the manuscript.
